# The Treatment of Rupture of the Kidney

**Published:** 1901-06-15

**Authors:** 


### THE TREATMENT OF RUPTURE OF THE KIDNEY.

According to some people rupture of the kidney should be treated by nephrectomy. In an address delivered before the Cardiff Medical Society last week Mr. Edmund Owen showed on the contrary that, with a little assistance, on ordinary surgical principles, Nature can deal very satisfactorily with such cases. He pointed out that, in regard to safety of position in the abdomen, the kidneys come next to the aorta and vena cava, lying as they do close by the side of the vertebral column upon the lower ribs and the diaphragm, and comfortably packed in the midst of a quantity of semi-fluid fat. There, if anywhere, they ought to be safe from injury. But a kick from a horse, or a blow from the shaft of a cart, is apt to find them out, and rupture their somewhat friable tissue. Hæmorrhage is the inevitable result of rupture of the kidney, some of the blood finding its way down the ureter and giving rise to hæmaturia, and probably still more of it being extravasated into the loose connective tissue between the transversalis fascia and the peritoneum, for as a rule in these cases the peritoneum is not torn. If a large division of the renal artery be lacerated the bleeding may be very serious, the patient becoming blanched and a definite dulness occurring in the lumbar region. But a tumour in the loin arising in association with a rupture of the kidney is not always due to hæmorrhage, for it often happens that the hæmaturia clears up and that aseptic urine quickly collects in the perinephritic tissue with scarcely a stain of blood. The outlook is, of course, most serious when the injury is quickly followed by the formation of a lumbar swelling, and when the increased collapse points to internal hæmorrhage.

In illustration of his point Mr. Owen related a case of a boy of seven who was kicked by a horse on the front of the left side of the abdomen. He immediately became blanched, but complained of no great pain. No abrasion could be found, but the muscles of the abdomen were tense and tender. Beyond this nothing abnormal was discovered, but persistent vomiting supervened, lasting 24 hours. In the evening a considerable quantity of blood was passed with the urine, and this continued for two days, when the urine became free from blood. Seventeen days later a tumour the size of an orange was made out in the left lumbar region, which rapidly increased. After another 10 days or so this tumour was aspirated, a wine-bottleful of bloody urine being withdrawn, and in two or three days the same thing had to be repeated with the same result. Mr. Owen therefore advised that an incision should be made in the loin for exploration and drainage. This being done the kidney was found torn across the upper and lower portions being separated by a considerable space in which blood-stained urine had collected. The capsule of the kidney had been torn right across, and blood and urine had evidently collected in the loose tissue around the kidney and between the fragments. A drainage tube was placed in the wound, and the boy made a complete recovery.

Commenting upon this case, Mr. Owen said that he wished to call attention rather strongly to the fact that because a kidney is ruptured it need not necessarily be removed, because he had seen it stated in more places than one that nephrectomy was the line of treatment to be adopted if a kidney were found to be ruptured, and because triumphant reports every now and then appeared of cases thus ruthlessly dealt with. He felt sure that such radical and heroic treatment would never have been thought of, much less advised, if human subjects had been supplied only with one kidney. But because Nature had been bountiful should surgery become reckless? He was satisfied that if, on making a lumbar incision for the treatment of hæmorrhage either directly after the receipt of the injury or later on for dealing with the hydronephrosis, the kidney were found to be torn across, Nature who is resourceful as well as bountiful should be afforded the opportunity of showing how much she would be able to effect for the repair of that damaged organ. Then if, after all, the persistence of a renal fistula showed that the clearing of the ureter of clot or the efficient repair of the kidney could not be effected, nephrectomy might be thought of. But even then it should not be resorted to until everything had been tried with a view of restoring efficiency, always bearing in mind, of course, the dangers attaching to prolonged suppuration. But suppose that on making a lumbar incision it were found that not only was the capsule ruptured, but that the rent extended into the peritoneum, so that hæmorrhagic and urinary extravasation had taken place into the abdominal cavity, the patient could easily be placed in the supine position, the peritoneal cavity opened and wiped out, and a drain conveniently arranged through the loin wound. Even in such a case, unless the kidney were hopelessly damaged, the desire of the operator should be to afford it a chance of undergoing repair.

